# Treatment of Chronic Gastritis with Traditional Chinese Medicine: Pharmacological Activities and Mechanisms

**DOI:** 10.3390/ph16091308

**Published:** 2023-09-15

**Authors:** Lisheng Chen, Shizhang Wei, Yong He, Xin Wang, Tingting He, Aozhe Zhang, Manyi Jing, Haotian Li, Ruilin Wang, Yanling Zhao

**Affiliations:** 1 School of Pharmacy, Chengdu University of Traditional Chinese Medicine, Chengdu 611137, China; chenlsls27@163.com (L.C.); heffly2000@hotmail.com (Y.H.); wangxin1214069407@163.com (X.W.); jingmanyi@yeah.net (M.J.); swordsman_0526@163.com (H.L.); 2 Department of Pharmacy, General Hospital of PLA, Beijing 100039, China; 3 Division of Integrative Medicine, The Fifth Medical Center, General Hospital of PLA, Beijing 100039, China; medicinett@163.com (T.H.); aozhezhang@outlook.com (A.Z.); wrl7905@163.com (R.W.)

**Keywords:** chronic gastritis, classical prescription, proprietary Chinese medicine, active components, mechanism

## Abstract

Chronic gastritis (CG) is a common clinical digestive system disease, which is not easyily cured and is prone to recurrence. Traditional Chinese medicine (TCM) plays a significant role in the treatment of CG and has attracted increasing attention for clinical applications. In recent years, a large number of reports have shown that TCM has good therapeutic effect on CG. The aim of this paper is to investigate the pharmacological activities and mechanism of action of TCM in the treatment of CAG. Therefore, by searching the databases of Pubmed, China National Knowledge Infrastructure, Wanfang, and Baidu academic databases, this paper has summarized the molecular mechanisms of TCM in improving CG. The results show that the improvement of GC by TCM is closely related to a variety of molecular mechanisms, including the inhibition of *Helicobacter pylori* (Hp) infection, alleviation of oxidative stress, improvement of gastric function, repair of gastric mucosa, inhibition of inflammatory response, and apoptosis. More importantly, IRF8-IFN-γ, IL-4-STAT6, Hedgehog, pERK1/2, MAPK, PI3K-Akt, NF-κB, TNFR-c-Src-ERK1/2-c-Fos, Nrf2/HO-1, and HIF-1α/VEGF signaling pathways are considered as important molecular targets for TCM in the treatment of GC. These important findings will provide a direction and a basis for further exploring the pathogenesis of GC and tapping the potential of TCM in clinical treatment. This review also puts forward a bright prospect for future research of TCM in the treatment of CG.

## 1. Introduction

Chronic gastritis (CG) is the most common clinical disease of the digestive system, which refers to the gastric mucosal damage caused by the imbalance of aggressive and defensive factors [1]. Its essence is that the gastric mucosal structure changes after repeated damage to the gastric mucosal epithelium, which eventually leads to irreversible atrophy or even the disappearance of inherent gastric glands. According to statistics, the prevalence rate of CG in China is about 90% of patients undergoing gastroscopy [2]. The main symptoms of CG patients are epigastric discomfort, fullness, acid regurgitation, pain, poor diet, belching and other main symptoms. Some patients may have anxiety and depression. GC may occur at any age, which belongs to the category of “stomachache” and “ruffian fullness” in TCM [3]. At present, monotherapy or combination therapy is often used in the treatment of CG. There are many kinds of western medicine for the treatment of chronic gastritis, such as anti-gastric acid synthesis, eradication of Hp, anti-intestinal metaplasia and dysplasia, and protection of the gastric mucosa [4]. Due to the complex pathogenesis and mechanisms of CG, most western medicines only play a partial role in relieving symptoms, and the symptoms of CG are prone to relapse after withdrawal. In addition, western medicines also have serious adverse reactions.

**Figure 1:**
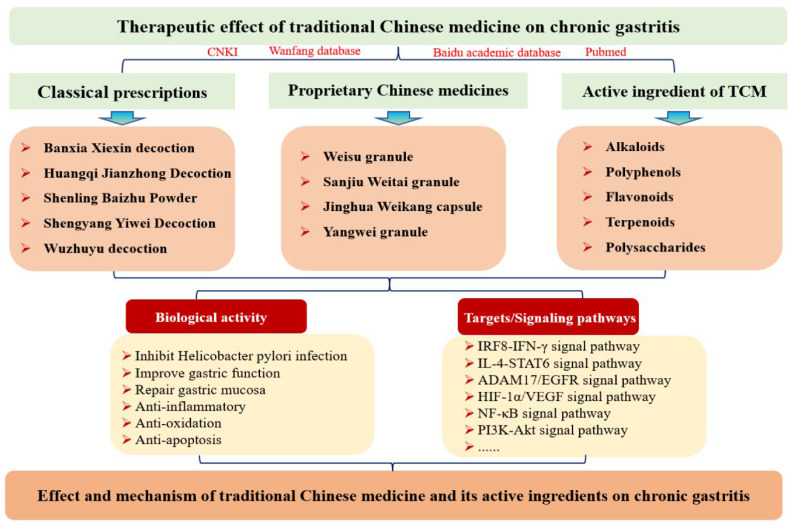
Flow chart of this study.

Given the relative poverty of modern integrative therapies, more and more researchers turn attention to TCM. In recent years, with the idea of integrated traditional Chinese and western medicine being put forward, the great curative effect of integrated traditional Chinese and western medicine in treating chronic gastritis has gradually attracted attention. The treatment of TCM has the characteristics of stable efficacy, high safety, low drug resistance, and low price, which not only has the advantage of relieving symptoms but also can significantly improve gastric mucosal injury and reduce the adverse reactions of modern comprehensive treatment. TCM emphasizes the overall concept of caring for the spleen and stomach, focusing on promoting the regulation of the body’s systemic immunity, protecting the local defense factors of the gastrointestinal mucosa, and improving the mucosal repair ability. TCM contains a variety of active ingredients with a wide range of pharmacological activities, representing a traditional combination therapy mode, which can well treat diseases and improve the quality of life of patients [5]. TCM contains a variety of components, which not only have a variety of biological activities but also may have special mechanisms of action. The best case supporting this idea is that artemisinin extracted from *Artemisia annua* can significantly improve the occurrence and development of malaria. At present, with the development of analytical chemistry and pharmacology, the study of pharmacological effects of natural components in traditional Chinese medicine has been rapidly developed, which provides a certain theoretical basis for the research and development of drugs [6,7]. The main purpose of this study is to review the latest research progress of TCM in the treatment of chronic gastritis. At the same time, the pharmacological effects and mechanism of the active components of related drugs were described in order to provide further inspiration for the development of new drugs for the treatment of CG and bring new hope to patients with chronic gastritis (Figure 1).

## 2. Classification of Chronic Gastritis

According to endoscopic and pathological diagnosis, chronic gastritis has been divided into non-atrophic (superficial) gastritis and atrophic gastritis [8]. Chronic superficial gastritis (CSG) is a common digestive system disease characterized by non-atrophic chronic inflammatory lesions of gastric mucosa. The common clinical endoscopic manifestations of CSG are mucosal erythema, mucosal bleeding spots or plaques, mucous membrane with or without edema, and hyperemia exudation [9,10]. According to epidemiological investigation, CSG occurs in all ages, accounting for about 42% to 78% of patients undergoing gastroscopy. More than 80% of adults have different degrees of CSG. Among them, the incidence of CSG is mostly in young and middle-aged people, and it gradually increases with age [11]. The development pattern of CSG lesions is “normal gastric mucosa—chronic non-atrophic gastritis—atrophic gastritis—intestinal metaplasia—dysplasia—gastric cancer” [12]. Clinical treatment of CSG in western medicine is mainly symptomatic treatment, mostly using antibiotics, proton pump inhibitors, colloidal bismuth drugs, acid suppressors, etc. [13]. However, there are certain adverse reactions; in addition, the course of treatment is long, and clinical recurrence is easy after withdrawal.

Chronic atrophic gastritis (CAG) is a chronic disease of the digestive system characterized by atrophy or reduction in inherent glands caused by damage to gastric mucosa epithelium and thinning of mucosal layer. Most patients have upper abdominal discomfort, fullness, gastric ulcer, and precancerous lesions [14]. As a common precancerous disease, CAG has an annual incidence of 10.9% worldwide, and its prevalence is always at a high level. In addition, the incidence of CAG is positively correlated with age [15]. Some studies have reported that the cancer rate of CAG in foreign countries is 8.6–13.8%, and that of CAG in China is 1.2–7.1% [16]. Therefore, the treatment of CAG is particularly important. At present, the treatment of CAG in modern medicine mainly aims to improve the symptoms of patients by eradicating Hp infection, enhancing barrier function, and reducing bile reflux [17,18,19]. However, it has the disadvantage of a high recurrence rate.

TCM has unique advantages and significant efficacy in the treatment of CG. TCM can carry out individualized treatment according to patients’ conditions, so that the patients’ physical conditions will be comprehensively adjusted.

## 3. Etiological Analysis of CG

A series of studies have shown that there are many pathogenic factors for chronic gastritis. The main reasons are heredity, constitution, immunity, diet and environment, Hp infection in the digestive tract, long-term use of drugs that stimulate the stomach (non-steroidal anti-inflammatory drugs, alcohol, etc.) and so on [20,21,22,23]. At present, it is found that Hp infection, diet, and environmental factors are the key factors that induce chronic gastritis. In recent years, with the rapid pace of people’s lives, long-term irregular diet, coupled with a large amount of smoking and alcoholism, not only increase the burden of the gastrointestinal tract but also bring serious damage to the gastric mucosa. At the same time, most people are in a state of stress at work for a long time, leading to different degrees of depression and mental stimulation. Under the influence of a variety of mental factors, abnormal secretion of gastric glands is induced, leading to CG [24,25,26].

*Helicobacter pylori* (Hp), a gram-negative bacterium, was first isolated in 1982 and selectively colonizes the gastric mucosa. It is one of the most common sources of infection in CG worldwide [27]. The prevalence of Hp infection varies greatly according to geographical region, age, race, and socio-economic status. The infection rate in developing countries seems to be higher than that in developed countries. It is estimated that approximately 50% of the world’s population is infected with Hp [28,29], with approximately one-third of adults in Northern and North America carrying Hp, and even more in Southern and Eastern Europe, South America, and Asia. This suggests that socio-economic status and the standard of living may play a significant role in infection [30].

Hp can last in a bad gastric environment for decades. It destroys the gastric mucosa and changes the release pattern of gastric hormones, thus affecting the physiological activity of the stomach [31]. After entering the stomach, Hp causes infection through the four steps of “living in acid stomach-moving to gastric epithelial cells-attaching host cells-causing tissue damage”, of which the first three steps determine the colonization of *Hp* in the stomach and play an important role in the process of the infection process [32]. Cytotoxicity-related genes protein A (CagA) and vacuolating toxin (VacA) are two cytotoxins produced by Hp that destroy gastric mucosa. Both of them damage epithelial cells by forming vacuoles, increasing cell permeability, changing the cytoskeleton, and activating related pathways to cause apoptosis [33,34]. In addition, Hp induces inflammation in gastric epithelial cells by up-regulating a variety of pro-inflammatory cytokines, such as NF-κB, IL-6, IL-8, and TNF-α [35]. If not treated in time, intestinal metaplasia and even gastric cancer will occur.

## 4. Clinical Observation of TCM in the Treatment of CG

### 4.1. Classical Prescriptions

Classical prescriptions are the outstanding representatives of traditional Chinese medicine prescriptions, summarizing the clinical experience of previous generations of medical practitioners. The classical prescription has characteristics of relatively stable ingredients, clear curative effects, profound historical and cultural accumulation, and so on. In addition, it possesses unparalleled advantages in theoretical basis, prescription laws, dose control, drug administration, and other aspects. It is an important carrier to reflect the clinical experience and academic thoughts of famous ancient doctors [5].

Banxia Xiexin decoction is a classic prescription of TCM, which was first recorded in the Treatise on Febrile Diseases in the Medical Code of the Eastern Han Dynasty. This ancient prescription is composed of seven kinds of Chinese herbal medicine, including *Pinellia ternate* (Thunb.) Breit., *Coptis chinensis* Franch., *Scutellaria baicalensis* Georgi, *Zingiber officinale* Rosc., *Panax ginseng* C. A. Mey., *Ziziphus jujuba* Mill., *Glycyrrhiza uralensis* Fisch. In clinical practice, it has been widely used in the treatment of patients with the cold-heat syndrome of CAG. A recent study demonstrated that the clinical effective rate of Banxia Xiexin decoction in the treatment of chronic non-atrophic gastritis and improving gastritis symptoms is significantly better than that in the control group [36]. In addition, Banxia Xiexin decoction plays a more significant role in inhibiting Hp infection, improving Hp-related inflammation, reducing glandular atrophy, intestinal metaplasia, and gastric mucosal dysplasia [37]. It has been found that many kinds of Chinese herbs in this prescription can protect the gastric mucosa and inhibit Hp. In addition, it also relieves pain by inhibiting the central nervous system [5,38,39]. Numerous bioactive components in the above traditional Chinese medicines, including gingerol, soybean oil alcohol, stigmasterol, β-sitosterol, berberine, palmatine, baicalein, ginsenoside, polysaccharides and glycyrrhizin, have been found to play a direct or indirect role in the treatment of chronic gastritis [40].

Huangqi Jianzhong Decoction is derived from the Synopsis of the Golden Chamber written by Zhang Zhongjing in the Eastern Han Dynasty. It is composed of seven traditional Chinese medicines, including *Astragalus membranaceus* (Fisch.) Bge., *Cinnamomum cassia* Presl, *Paeonia lactiflora* Pall., *Zingiber officinale* Rosc., *Glycyrrhiza uralensis* Fisch., *Ziziphus jujuba* Mill., and *Saccharum* Granorum, which are used to treat various chronic inflammatory gastrointestinal diseases [41,42]. The study showed that Huangqi Jianzhong Decoction has a significant effect on the treatment of Hp-related gastritis with spleen and stomach deficiency and cold type. After treatment, nausea, vomiting, and upper abdominal discomfort were effectively improved, and the negative conversion rate of Hp was significantly increased [43]. In addition, in the treatment of chronic superficial gastritis, the efficacy was similar to that of omeprazole sodium in the improvement of stomachache, stomach distension, and nausea, with good safety, and no adverse reactions were found. In terms of effect duration, the effect of Huangqi Jianzhong Decoction was superior to that of the omeprazole treatment group [44]. In summary, the effect of Huangqi Jianzhong Decoction on chronic gastritis is outstanding, which is worthy of extensive clinical promotion.

Shenling Baizhu Powder comes from the “Prescription of peaceful benevolent dispensary,“ which consists of ten kinds of herbs: *Panax ginseng* C. A. Mey., *Poria cocos* (Schw.) Wolf, *Atractylodes macrocephala* Koidz., *Dioscorea opposita* Thunb., *Dolichos lablab* L., *Nelumbo nucifera* Gaertn., *Coix lacryma-jobi* L.var.ma-yuen (Roman.) Stapf, *Amomum villosum* Lour., *Platycodon grandiflorum* (Jacq.) A.DC., and *Glycyrrhiza uralensis* Fisch. [45]. The prescription has a bidirectional regulatory effect on gastrointestinal movement, which plays an important role in the treatment of gastric diseases. The evidence from this study suggested that Shenling Baizhu Powder can significantly relieve the clinical symptoms of patients with CAG and relieve gastric discomfort. It was mainly manifested in increasing the serum levels of PG Ⅰ, G-17, and PG Ⅱ, reducing gastric injury, and improving prognosis [46].

Shengyang Yiwei Decoction was first seen in “Internal and External Injury Discrimination · Volume,“ which is a famous prescription of Li Gao. The prescription consists of *Astragalus membranaceus* (Fisch.) Bge., *Pinellia ternate* (Thunb.) Breit., *Panax ginseng* C. A. Mey., *Glycyrrhiza uralensis* Fisch., *Paeonia lactiflora* Pall., *Saposhnikovia divaricata* (Turcz.) Schischk., *Notopterygium incisum* Ting ex H. T. Chang, *Angelica pubescens* Maxim.f. biserrata Shan et Yuan, *Citrus reticulata* Blanco, *Poria cocos* (Schw.) Wolf, *Alisma orientale* (Sam.) Juzep., *Bupleurum chinense* DC., *Atractylodes macrocephala* Koidz., and *Coptis chinensis* Franch. [47]. A study showed that patients with gastritis were treated with Shengyang Yiwei Decoction while receiving triple therapy of western medicine, which could significantly improve epigastric pain, belching, acid regurgitation, nausea, and vomiting, and improve the therapeutic effect and Hp clearance rate. The combination of the two can play a synergistic effect and is not easy to produce side effects [48].

Wuzhuyu Decoction was first seen in Zhang Zhongjing’s “Treatise on Febrile Diseases.” It is composed of *Euodia rutaecarpa* (Juss.) Benth., *Zingiber officinale* Rosc., *Panax ginseng* C. A. Mey., *Ziziphus jujuba* Mill., and has a good effect in the treatment of patients with stomach cold. In the treatment of gastric gastritis with liver cold invading stomach, through the comparison of Wuzhuyu Decoction and Rabeprazole capsule combined with aluminum-magnesium plus suspension, it was found that Wuzhuyu Decoction had a higher effective rate in the treatment of gastric gastritis with liver cold invading the stomach. In addition, the patient’s symptom improvement time is shortened, and the incidence of adverse reactions is lower [49] (Table 1).

### 4.2. Proprietary Chinese Medicines

Proprietary Chinese medicine is an important component in the clinical practice of TCM and plays an important role in global medical practice. It has the advantages of high bioavailability, convenient storage and probability, and controllable quality. In recent years, the increasing variety of proprietary Chinese medicine and the continuous innovation in dosage forms have led to their popularity worldwide.

Weisu granule is composed of *Perilla frutescens* (L.) Britt., Cyperus rotundus L., *Citrus reticulata* Blanco, *Citrus medica* L., *Citrus medica* L. var. sarco dactylis Swingle, *Citrus aurantium* L., *Areca catechu* L., *Gallus gallus domesticus* Brisson, which have the effects of anti-inflammation, eliminating Hp and improving gastric injury. There is evidence that Weisu granule combined with triple therapy can significantly improve gastric function, repair gastric mucosa, and reduce the level of inflammatory factors in patients with CG. Further studies have found that chuannoetin in tangerine peel has the effect of destroying bacterial structure [50]. Xiang Fu inhibits the production of inflammatory chemokines in order to inhibit the occurrence of inflammation and delay the development of the disease [51]. Sanjiu Weitai granule is mainly composed of *Evodia lepta* (Spreng.) Merr., *Scutellaria baicalensis* Georgi, *Murraya exotica* L., *Zanthoxylum nitidum* (Roxb.) DC., *Aucklandia lappa* Decne., *Poria cocos* (Schw.) Wolf, *Paeonia lactiflora* Pall., *Rehmannia glutinosa* Libosch. The combined application of various herbs plays the role of clearing heat, removing dampness, reducing inflammation and relieving pain. A study of Sanjiu Weitai granule combined with rabeprazole sodium enteric-soluble capsule in the treatment of chronic superficial gastritis found that the clinical effective rate of the combined application group was significantly higher than that of rabeprazole sodium enteric-soluble capsule alone, and the incidence of adverse reactions was reduced [52]. Jinghua Weikang capsule is a kind of capsule made from volatile oil extracted from *Adina pilulifera* (Lam.) Franch et Drake, and *Chenopodium ambrosioides* L by modern pharmaceutical technology under the guidance of traditional Chinese medicine theory. It is found that Jinghua Weikang capsule has a good anti-Hp effect in vivo and in vitro, which has a certain repair and protective effect on gastric mucosal injury caused by Hp. Its mechanism may be related to the inhibition of NF-κB-mediated inflammatory pathway. Moreover, it also has a certain effect in improving drug resistance [53]. Qi et al. [54] discussed the clinical efficacy of Jinghua Weikang capsule combined with Rebaport tablets in the treatment of CAG. The results showed that the total effective rate, prostaglandin E2 (PGE2), gastrin-17 (G-17), pepsinogen ratio (PGR) and pepsinogen Ⅰ (PG Ⅰ) in Jinghua Weikang capsule combined with Rebapide tablets were higher than those in the group treated with Rebapide tablets alone. In addition, the levels of serum soluble interleukin-2 receptor (SIL-2R) and interleukin-1 β (IL-1 β) were lower than those in the treatment group treated with Ribapide alone, indicating that Jinghua Weikang capsule combined with Rebapite tablet can improve the curative effect of chronic atrophic gastritis, reduce the inflammatory injury of gastric mucosa and regulate gastrointestinal hormone secretion with good safety and good clinical application. Yangwei granule is composed of *Astragalus membranaceus* (Fisch.) Bge., *Codonopsis pilosula* (Franch.)Nannf., *Paeonia lactiflora* Pall., *Glycyrrhiza uralensis* Fisch., *Citrus reticulata* Blanco, *Cyperus rotundus* L., *Prunus mume* (Sieb.) Sieb.etZucc., and *Dioscorea opposita* Thunb. Modern pharmacological studies have found that it can reduce pepsin activity, inhibit gastric acid secretion, improve gastric motility and increase pain threshold [55,56]. The study has shown that Yangwei granule combined with anti-HP quadruple therapy relieves the clinical symptoms of CAG with Hp infection more quickly than quadruple therapy alone. In addition, Yangwei granule combined with anti-HP quadruple therapy significantly improves the clinical efficacy and Hp eradication rate with good safety and high clinical value [57] (Table 2).

## 5. Active Ingredient of TCM in Treating Chronic Gastritis

### 5.1. Alkaloid

Alkaloids are basic organic compounds containing nitrogen in plants. They are one of the important effective components in Chinese herbal medicine, and most of them have complex ring structures. Studies have shown that alkaloids have a wide range of pharmacological activities, which protect gastric injury through anti-inflammation, anti-oxidation, anti-apoptosis, protection of gastric mucosa, antibacterial, and more. Berberine and palmatine are natural isoquinoline alkaloids extracted from Coptis chinensis and other Chinese herbal medicines, and they have been widely used in treatment for hundreds of years. In the past few decades, many studies have shown that berberine and palmatine and their derivatives have anti-inflammatory, bacteriostatic, and mucosal protection activities in the digestive system [58,59]. At the same time, more and more reports have clarified that they have great potential in the treatment of CG caused by HP infection. Yang and his colleagues’ investigation indicated that berberine had a good therapeutic effect on CAG caused by Hp [60,61]. Its mechanism might be partly related to the inhibition of interferon regulatory factor 8 (IRF8)-interferon-γ (IFN-γ) signal axis and the activation of interleukin-4 (IL-4)-signal transducer and activator of transcription 6 (STAT6) signal pathway. Under the intervention of berberine, serum interleukin-17 (IL-17), chemokine 1(CXCL1) and chemokine 9 (CXCL9) levels were down-regulated, while G-17 was significantly increased. The damage of gastric mucosa caused by Hp was also alleviated. At the same time, berberine could improve the cell viability and morphological changes of GES-1 cells. On the other hand, berberine treatment inhibited pro-inflammatory genes and IRF8-IFN-γ signal axis related genes. In addition, the mRNA expression of tumor necrosis factor-α (TNF-α), iNOS (NOS2), chemokine receptor type 7 (CCR7), and IRF-8 were suppressed and the mRNA expression of IL-4, STAT6, and interleukin-10 (IL-10) were increased by berberine intervention. Another study revealed that palmatine significantly attenuated Hp-induced gastric mucosal injury and morphological changes in GES-1 cells. Further research indicated that palmatine significantly inhibited the expression of EGFR-activated ligand genes, including a decomposed and metalloproteinase 17 (ADAM17) and heparin-bound epidermal growth factor-like growth factor (HB-EGF). In addition, palmatine attenuated the inflammatory infiltration of CD8+T cells and inhibit chemokine 16 (CXCL-16) and interleukin 8 (IL-8), thus enhancing host defense [62]. Dehydroevodiamine, a key quinazoline alkaloid isolated from *Evodia rutaecarpa*, plays an important role in the treatment of gastrointestinal diseases. The results of Wen’s team suggested that dehydroevodiamine ameliorated MNNG-induced gastric injury in CAG rats in vivo and GES-1 cell migration in vitro by inhibiting HIF-1α/VEGF angiogenesis pathway [63].

### 5.2. Polyphenols

Polyphenols are complex secondary metabolites with multiple phenolic hydroxyl groups, widely distributed in plants, and have pharmacological effects such as antioxidation, free radical scavenging, analgesia, anti-inflammation, and antibacterial. Increasing evidence indicate that polyphenol compounds have certain therapeutic and ameliorative effects on gastric injury. Turmeric, a subtropical plant belonging to the ginger family, contains curcumin, a major component contributing to its many biological functions. Studies have shown that curcumin has a wide range of biological functions, such as anti-inflammatory, antioxidant, antimicrobial, neuroprotective, and gastric protective properties [64,65]. Research suggested that curcumin could protect and heal the stomach by increasing the expression of matrix metalloproteinase-2 (MMP-2) and reducing the activity of matrix metalloproteinase-9 (MMP-9), resulting in re-epithelialization and remodeling of mucous membrane [66]. In addition, Hp-induced gastric inflammation was associated with increased NF-κB activation. The NF-κB pathway participates in the production of these inflammatory mediators (TNF-α, IL-6, and IL-1β), which were reduced by curcumin supplementation. Additionally, curcumin decreased the expression of a set of chemokines (CCL20, CCL5, CXCL1, CXCL10, CXCL11, CCL25). Furthermore, Hp activates several TLRs on epithelial and dendritic cells (DCs). TLRs are key molecules mediating the interaction between Hp and DCs, which is largely dependent on the adaptor protein MyD88 signaling that is used by all TLRs except TLR3. Activated TLR4 activates NF-κB through the MyD88-dependent pathway to regulate the production of inflammatory cytokines. Curcumin decreased the expression of TLRs and MyD88, thus exhibiting anti-inflammatory effect. The mechanism were partially recognized as improving the expression of inflammatory cytokines, chemokines as well as toll-like receptors (TLRs) and myeloiddifferentiationfactor88 (MyD88) in Hp-induced gastritis mice [67]. Clinically, compared with the triple therapy group, the curcumin combined with triple therapy group significantly decreased the markers of malondialdehyde and glutathione peroxidation products, and improved the total antioxidant capacity of gastric mucosa. Furthermore, the oxidative damage of DNA in the curcumin combined with triple therapy group was significantly lower than that in the baseline and triple therapy groups. Further research shows that the scores of all active inflammation, chronic inflammation and gastroscopy inflammation in the curcumin combined with triple therapy group were significantly lower than those in the triple therapy group [68]. Resveratrol, mainly derived from *Polygoni Cuspidati Rhizoma et Radix*, is a naturally occurring phytonutrient polyphenolic compound used to treat a variety of ailments, including pain, tissue damage, and inflammatory diseases. It is well known that resveratrol can regulate the protein expression of various virulence factors induced by Hp. The mechanism of this activity is partially related to inhibiting the levels of IL-8 and iNOS in Hp-induced gastritis model by up-regulating nuclear factor erythroid2-related factor 2 (Nrf2)/heme oxygenase-1(HO-1) signal pathway [69].

### 5.3. Flavonoids

Flavonoids are widely found in all kinds of plants and usually combine with sugars to form glycosides. A large number of studies have found that flavonoids have a variety of pharmacological activities, such as anti-gastric ulcer, anti-inflammation, antioxidation, and analgesia. It was found that quercetin decreased the expression of MMP-9 in GES-1 cells induced by TNF-α and protected gastric mucosal epithelial cells by regulating TNFR-c-Src-ERK1/2-c-Fos and NF-κB pathway [70]. Another study further revealed that kaempferol could play a role in the treatment of chronic atrophic gastritis by regulating the Hedgehog signal pathway and reducing the levels of IL-6 and IL-1β, which provide a theoretical basis for revealing the treatment of chronic atrophic gastritis with kaempferol [71]. Baicalin is a natural flavonoid compound with antioxidant, anti-inflammatory, and immunomodulatory activities. Ji et al. reported that baicalin participated in several pathways such as mitogen-activated protein kinase (MAPK), phosphatidylinositol 3 kinase (PI3K)-Akt, and NF-κB to reduce the production of IL-2, IL-8, and TNF-α, and to increase the expression of epidermal growth factor and b-cell lymphoma-2. The above results provide important insights into the discovery of potential target proteins for the treatment of CG [72].

### 5.4. Terpenoids

Terpenoids are important natural organic compounds contained in TCM, which are composed of isoprene as the basic structural unit. Terpenoids play an important role in treating gastritis by regulating related signal pathways, improving inflammation and gastric mucosa injury. Gentiopicrin is the main active ingredient of gentian. It was found that treatment with gentiopicrin significantly improved alcohol-induced gastritis in mice, mainly by decreasing the production of pro-inflammatory cytokines TNF-α, IL-1β, and IL-8, and increasing the level of anti-inflammatory cytokine IL-10. This research finally found that regulating the MMP-10 and pERK1/2 signaling pathway is the crucial mechanism of gentiopicroside in alcohol-induced gastritis [73]. Ginsenoside Rg1 is the main active ingredient of Panax notoginseng with the highest content. A study demonstrated that ginsenoside Rg1 could improve the pathological condition of chronic atrophic rats and effectively inhibit the occurrence of atrophy and inflammation. Furthermore, ginsenoside Rg1 increased the expression of SHH and Ptch in gastric tissue, suggesting that ginsenoside Rg1 may improve gastric mucosal lesions in CAG by activating the Hedgehog pathway [74].

### 5.5. Polysaccharides

**Figure 2:**
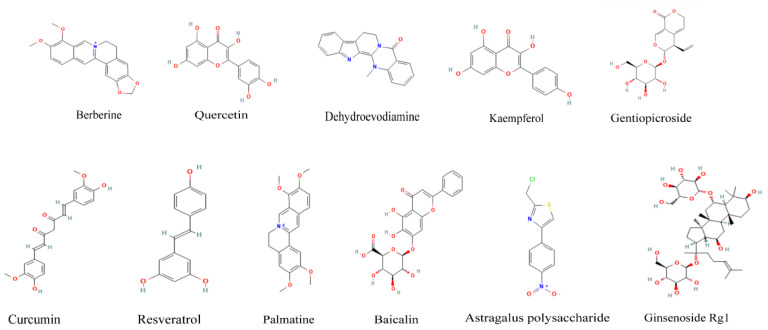
Chemical structure of active components of traditional Chinese medicine. Note: these chemical structural formulas are derived from PubChem.

Polysaccharides widely exist in a variety of Chinese herbal medicine, which have a significant effect on the protection of gastric mucosal damage. They are characterized by multi-angle and multi-institutional coordination. Astragalus polysaccharides, as the main active component of Astragalus membranaceus, are one of the important material bases of its pharmacological action. It was found that astragalus polysaccharides have a certain therapeutic effect on CAG rats by down-regulating cyclooxygenase-2 (COX-2) and MMP2. The gastric morphology of chronic non-atrophic gastritis rats was significantly improved after treatment with astragalus polysaccharide. In addition, astragalus polysaccharide significantly increased the levels of gastrin in plasma and decreased PGE2 in CAG rats but had no significant effect on motilin levels [75]. Glycyrrhiza polysaccharides are one of the main components of *Glycyrrhiza uralensis Fisch.* The research found that the significant pharmacological effects of antioxidation and anti-apoptosis were related to increasing the ratio of Bcl-2/Bax and the content of SOD and GSH in gastric mucosa [76] (Table 3, Figure 2).

## 6. Conclusions and Perspectives

**Figure 3:**
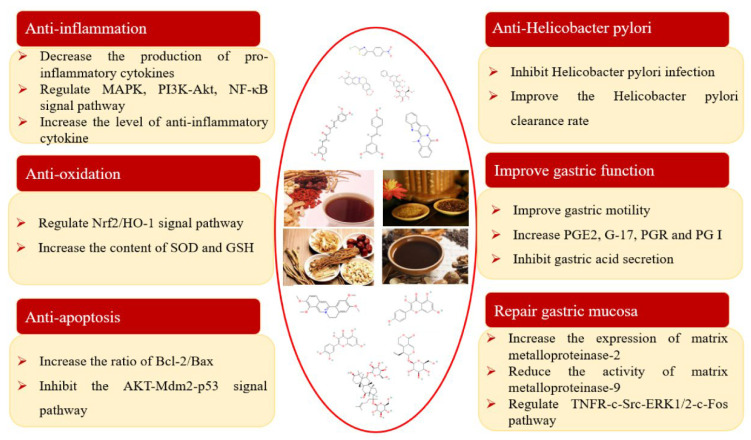
The pharmacological properties of traditional Chinese medicine and its main active components on CG.

**Figure 4:**
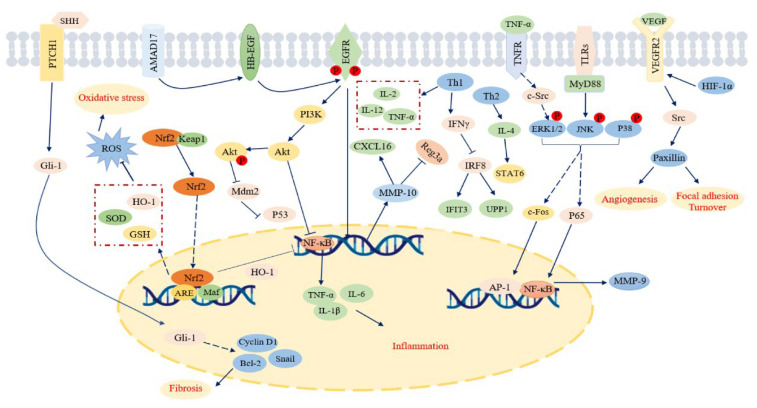
The possible molecular mechanisms of main active components on CG.

With the emergence of western drug resistance, the effect of western medicine targeted drugs in the treatment of CG has been affected in varying degrees, and the failure rate of treatment is increasing year by year. In recent years, TCM has made remarkable progress in the prevention and treatment of chronic gastritis. Many classical prescriptions, proprietary Chinese medicines, and active components of TCM have shown significant therapeutic effects in pharmacological experiments and clinical applications. The study summarizes the importance of classical prescriptions, proprietary Chinese medicines, and active ingredients of TCM in the treatment of CG. It is also crucial to reveal the mechanism to determine how classical prescriptions, proprietary Chinese medicines, and active ingredients of TCM exert their pharmacological effect. Studies have found that classical prescriptions, proprietary Chinese medicines, and active ingredients of traditional Chinese medicine can treat chronic gastritis through anti-inflammation, anti-oxidation, anti-apoptosis, immune regulation, inhibition of Hp infection, protection of gastric mucosa, pain relief, antiemetic, and so on. Further research indicated that the mechanism of these activities are partially related to IRF8-IFN-γ, IL-4-STAT6, Hedgehog, pERK1/2, MAPK, PI3K-Akt, NF-κB, TNFR-c-Src-ERK1/2-c-Fos, Nrf2/HO-1, and HIF-1α/VEGF signaling pathways (Figure 3 and Figure 4).

However, there are still many problems to be solved in the treatment of CG with TCM. Firstly, the mechanism of TCM in the treatment of CG has not been fully revealed, and the relationship between the pathways are not clear. Therefore, the evaluation of the correlation between these pathways may help to provide a more reliable basis for understanding the mechanism of TCM in the treatment of CG. Secondly, it is necessary to further clarify the effective components of TCM in the treatment of CG, strengthen the research on the compatibility between the effective components and TCM, and pay attention to the combination of pharmacological experiments and clinical verification. Although pharmacological studies have shown that most of the active ingredients of TCM are safe and effective, most of the active ingredients of TCM have not been developed and applied in the clinic. The toxicological studies of the active components of TCM need to be further improved and supplemented. In addition, determining the optimal clinical dose of some TCM active ingredients to play a role in the treatment of chronic gastritis needs to be addressed. Therefore, special attention should be paid to the “basic-clinical” transformation. Further mechanism research and further clinical confirmation are still two key processes for the future development of TCM. Thus, comprehensively analyzing the biological information data of multiple dimensions through the multi-omics study of TCM can help to comprehensively understand the efficacy and mechanism of TCM, aiding researchers to explore the relationship between various components of TCM, revealing their interaction mechanisms, and regulating disease targets. This will lead to a better understanding of the overall pharmacological effects of TCM.

## Figures and Tables

**Table 1:** The pharmacological activities of classical prescription in CAG.

Prescription	Source	Composition	Pharmacological Action	Reference
Banxia Xiexin decoction	Treatise on Febrile Diseases	*Pinellia ternate* (Thunb.) Breit.(15 g), *Coptis chinensis* Franch.(9 g), *Scutellaria baicalensis* Georgi(3 g), *Zingiber officinale* Rosc. (9 g), *Panax ginseng* C. A. Mey. (9 g), *Ziziphus jujuba* Mill. (9 g), *Glycyrrhiza uralensis* Fisch.(4 grains).	(1) Inhibit Hp infection; (2) Improve Hp-related inflammation; (3) Reduce glandular atrophy; (4) Intestinal metaplasia and gastric mucosal dysplasia; (5) Reduce the incidence of adverse reactions.	[37]
Huangqi Jianzhong decoction	Synopsis of the Golden Chamber	*Astragalus membranaceus* (Fisch.) Bge.(45 g), *Cinnamomum cassia* Presl(90 g), *Paeonia lactiflora* Pall. (180 g), *Zingiber officinale* Rosc.(90 g), *Glycyrrhiza uralensis* Fisch.(60 g), *Ziziphus jujuba* Mill.(12 grains), *Saccharum* Granorum(1 L).	(1) Increase the negative conversion rate of Hp; (2) Improve stomachache, stomach distension and nausea; (3) Improve epigastric discomfort.	[43]
Shenling Baizhu Powder	Prescription of peaceful benevolent dispensary	*Panax ginseng* C. A. Mey. (100 g), *Poria cocos* (Schw.) Wolf (100 g), *Atractylodes macrocephala* Koidz. (100 g), *Dioscorea opposita* Thunb. (100 g), *Dolichos lablab* L. (75 g), *Nelumbo nucifera* Gaertn. (50 g), *Coix lacryma-jobi* L.var.ma-yuen (Roman.) Stapf (50 g), *Amomum villosum* Lour. (50 g), *Platycodon grandiflorum* (Jacq.)A.DC. (50 g), *Glycyrrhiza uralensis* Fisch. (100 g).	(1) Bidirectional regulation of gastrointestinal movement; (2) Improve gastrointestinal function; (3) Relieve the patient’s stomach discomfort; (4) Reduce gastric injury; (5) Improve prognosis.	[46]
Shengyang Yiwei decoction	Internal and External Injury Discrimination · Volume	*Astragalus membranaceus* (Fisch.)Bge. (30 g), *Pinellia ternate* (Thunb.) Breit. (15 g), *Panax ginseng* C. A. Mey. (15 g), *Glycyrrhiza uralensis* Fisch. (15 g), *Paeonia lactiflora* Pall. (9 g), *Saposhnikovia divaricata* (Turcz.) Schischk. (9 g), *Notopterygium incisum* Ting ex H. T. Chang (9 g), *Angelica pubescens* Maxim.f. biserrata Shan et Yuan(9 g), *Citrus reticulata* Blanco (6 g), *Poria cocos* (Schw.)Wolf (5 g), *Alisma orientale* (Sam.)Juzep. (5 g), *Bupleurum chinense* DC. (5 g), *Atractylodes macrocephala* Koidz. (5 g), *Coptis chinensis* Franch. (1.5 g).	(1) Improve epigastric pain, belching, acid regurgitation, nausea and vomiting, fatigue; (2) Improve the therapeutic effect and Hp clearance rate; (3) Improve prognosis.	[48]
Wuzhuyu decoction	Treatise on Febrile Diseases	*Euodia rutaecarpa* (Juss.) Benth. (9 g), *Zingiber officinale* Rosc. (18 g), *Panax ginseng* C. A. Mey. (9 g), *Ziziphus jujuba* Mill.(12 grains).	(1) Warming the stomach and dispelling cold; (2) Reducing adverse reaction and stopping vomiting; (3) Replenishing qi and invigorating spleen.	[49]

**Table 2:** The pharmacological activities of proprietary Chinese medicine in CAG.

Proprietary Chinese Medicine	Composition	Pharmacological Action	Reference
Weisu granule	*Perilla frutescens* (L.) Britt., Cyperus rotundus L., *Citrus reticulata* Blanco, *Citrus medica* L., *Citrus medica* L. var. sarco dactylis Swingle, *Citrus aurantium* L., *Areca catechu* L., *Gallus gallus domesticus* Brisson	(1) Improve gastric function; (2) Repair gastric mucosa; (3) Reduce the level of inflammatory factors in patients; (4) Improve the efficiency of clinical treatment; (5) Elimination of Hp.	[50]
Sanjiu Weitai granule	*Evodia lepta* (Spreng.) Merr., *Scutellaria baicalensis* Georgi, *Murraya exotica* L., *Zanthoxylum nitidum* (Roxb.) DC., *Aucklandia lappa* Decne., *Poria cocos* (Schw.) Wolf, *Paeonia lactiflora* Pall., *Rehmannia glutinosa* Libosch.	(1) Clearing heat and dampness; (2) Eliminating inflammation; (3) Relieving pain; (4) Regulating qi and eliminating distension; (5) Reducing the incidence of adverse reac-tions.	[52]
Jinghua Weikang capsule	*Chenopodium ambrosioides L*, *Adina rubella Hance*	(1) Anti- Hp (2) Inhibition of inflammatory pathway mediated by NF-κB; (3) Improve drug resistance; (4) Regulation of gastrointestinal hormone secretion; (5) Improve the curative effect of gastritis.	[53,54]
Yangwei granule	*Astragalus membranaceus* (Fisch.) Bge., *Codonopsis pilosula* (Franch.) Nannf., *Paeonia lactiflora* Pall., *Glycyrrhiza uralensis* Fisch., *Citrus reticulata* Blanco, *Cyperus rotundus* L., *Prunus mume* (Sieb.) Sieb.etZucc., *Dioscorea opposita* Thunb.	(1) Reduce pepsin activity (2) Inhibit gastric acid secretion; (3) Increase gastric motility and pain threshold; (4) Improve clinical efficacy; (5) Improve HP eradication rate.	[57]

**Table 3:** The pharmacological activities of active components of traditional Chinese medicine in CAG.

Component Classification	Component	Animal/Cell Model	Doses	Targets/Pathways	Reference
Alkaloids	Berberine	Hp-infected rats with CAG; Hp or LPS-infected GES-1/RAW 264.7 cells	14, 28 mg/kg; 20, 40 μM	IRF8-IFN-γ pathway; IL-4-STAT6 pathway.	[60,61]
	Palmatine	Hp-infected rats with CAG; Hp-infected GES-1 cells	10, 20 mg/kg; 20, 40 μM	ADAM17/EGFR pathway	[62]
	Dehydroevodiamine	MNNG-induced CAG rats/GES-1 cells	5, 10 mg/kg; 2.5, 5, 10μM	HIF-1α/VEGF pathway	[63]
Polyphenols	Curcumin	Indomethacin-induced gastric ulcer in rats; Hp-infected mice with CAG	60 mg/kg; 500 mg/kg	MMP-2, TGF-β, and VEGF; TLRs, MyD88	[66,67]
	Resveratrol	Hp-induced mice with gastric inflammation	100 mg/kg	Nrf2/HO-1 pathway, IL-8, iNOS, and NF-κB	[69]
Flavonoids	Quercetin	TNF-α-induced GES-1 cells	0.1, 0.3, 1μM	TNFR-c-Src-ERK1/2-c-Fos pathway, NF-κB pathway,	[70]
	Kaempferol	CAG rats	50 mg/kg	Hedgehog pathway	[71]
	Baicalin	56% ethanol-induced chronic gastritis in rats	50 mg/kg	MAPK, PI3K-Akt, and NF-κB pathway	[72]
Terpenoids	Gentiopicroside	Ethanol-induced gastritis	50, 100 mg/kg	MMP-10 and pERK1/2 pathway	[73]
	Ginsenoside Rg1	Atrophic gastritis induced by pyloric spring in rats	0.5, 1 mg/kg	Hedgehog pathway	[74]
Polysaccharides	Astragalus polysaccharides	MNNG-induced CAG rats	0.5, 1 g/kg	EGFR, COX-2 and MMP2	[75]
	Licorice polysaccharide	Ethanol-induced chronic gastritis in rats	1 g/kg	Bcl-2/Bax, SOD and GSH	[76]

## Data Availability

Data sharing is not applicable.

## Abbreviations

CG	Chronic gastritis	NF-κB	Nuclear factor kappa B
TCM	Traditional Chinese medicine	IL-6	Interleukin-6
CSG	Chronic superficial gastritis	IL-8	Interleukin-8
CAG	Chronic atrophic gastritis	TNF-α	Tumor necrosis factor alpha
Hp	Helicobacter pylori	PGE2	Prostaglandin E2
CagA	Cytotoxicity-related genes protein A	G-17	Gastrin-17
VacA	Vacuolating toxin	PGR	Pepsinogen ratio
SIL-2R	Soluble interleukin-2 receptor	PG Ⅰ	Pepsinogen Ⅰ
IL-4	Interleukin-4	IL-1β	Interleukin-1β
IL-10	Interleukin-10	IL-17	Interleukin-17
IRF8	Interferon regulatory factor 8	CXCL1	C-X-C Motif Chemokine Ligand 1
IFN-γ	Interferon-gamma	CXCL9	C-X-C Motif Chemokine Ligand 9
CCR7	C-C motif chemokine receptor 7	NOS2	Nitric oxide synthase 2
IL-8	Interleukin-6	EGFR	Epidermal Growth Factor Receptor
HIF-1α	Hypoxia-inducible factor-1 alpha	MyD88	Myeloiddifferentiationfactor88
VEGF	Vascular endothelial growth factor	iNOS	Inducible nitric oxide synthase
TLRs	Toll-like receptors	CXCL-16	Chemokine 16
MMP-9	Matrix metallopeptidase-9	HO-1	Heme oxygenase-1
TNFR	Tumor necrosis factor receptor	ADAM17	Metalloproteinase 17
MAPK	Mitogen-activated protein kinase	PI3K	Phosphoinositide 3-kinase
IL-2	Interleukin-2	Akt	Protein kinase B
COX-2	Cyclooxygenase-2	MMP2	Matrix metallopeptidase-2
Bcl-2	B-cell lymphoma-2	SHH	Sonic Hedgehog
SOD	Superoxide dismutase	GSH	Glutathione
HB-EGF	Heparin-bound epidermal growth factor-like growth factor	ERK1/2	Extracellular signal-regulated kinase 1/2
STAT6	Signal transducer and activator of transcription 6	Nrf2	Nuclear factor erythroid 2-related factor 2
